# Monitoring neoadjuvant treatment-induced surgical benefit in GIST patients using CT-based radiological criteria

**DOI:** 10.1016/j.sopen.2024.07.002

**Published:** 2024-07-14

**Authors:** Ylva A. Weeda, Gijsbert M. Kalisvaart, Henk H. Hartgrink, Aart J. van der Molen, Hans Gelderblom, Judith V.M.G. Bovée, Lioe-Fee de Geus-Oei, Willem Grootjans, Jos A. van der Hage

**Affiliations:** a Department of Radiology, Leiden University Medical Center, 2333 ZA Leiden, the Netherlands; b Department of Surgical Oncology, Leiden University Medical Center, 2333 ZA Leiden, the Netherlands; c Department of Medical Oncology, Leiden University Medical Center, 2333 ZA Leiden, the Netherlands; d Department of Pathology, Leiden University Medical Center, 2333 ZA Leiden, the Netherlands; e Biomedical Photonic Imaging Group, University of Twente, 7522 NB Enschede, the Netherlands; f Department of Radiation Science & Technology, Delft University of Technology, 2629 JB Delft, the Netherlands

**Keywords:** Gastrointestinal stromal tumour, Response monitoring, Tyrosine kinase inhibitors, Computed tomography, Surgery

## Abstract

**Objective:**

This single-centre retrospective study aims to determine the incidence of therapy-induced surgical benefit in patients with non-metastatic gastrointestinal stromal tumour (GIST) treated with neoadjuvant tyrosine kinase inhibitors (TKI) and evaluate whether this can be predicted by radiological response criteria.

**Methods:**

Thirty-nine non-metastatic GIST patients were treated with neoadjuvant TKI treatment, followed by curative-intended surgery, and monitored using contrast-enhanced computed tomography (CE-CT). Surgical benefit was independently assessed by two surgical oncologists and was defined by de-escalation of surgical strategy or reduced surgical complexity. Radiological response between baseline and the last preoperative scan was determined through RECIST 1.1, Choi and volumetric criteria.

**Results:**

In this patient cohort, median neoadjuvant treatment interval was 8.3 (IQR, 3.9–10.6) months. Surgical benefit was gained in 22/39 patients. When comparing radiological criteria to findings on surgical benefit, accuracy, sensitivity, and specificity for RECIST 1.1 (90 %, 100.0 % and 82 %), Choi (64 %, 24 %, and 96 %) and volumetry (95 %, 100.0 %, and 91 %) were calculated. In 30/39 patients, temporal changes in tumour size over the course of treatment was assessed. Tumour volume reduced significantly in the surgical-benefit group compared to the non-benefit group (72 % vs. 25 %, *p* < 0.01) within three months. 14/19 surgical-benefit patients had an initial volume reduction above 66 %, after which volume reduced slightly with a median 3.1 % (IQR, 2.1–7.8 %) reduction.

**Conclusion:**

Surgical benefit after neoadjuvant treatment was achieved in 56 % of patients and was most accurately reflected by size-based response criteria. In patients with therapy-induced surgical benefit, nearly all treatment-induced volume reductions were achieved within three months.

## Introduction

Gastrointestinal stromal tumour (GISTs) is a rare mesenchymal neoplasm with a worldwide incidence of 1–2 per 100,000 [1,2]. In localised disease, neoadjuvant TKI treatment is administered in a selected group of patients to increase probability of complete surgical excision, while sparing surrounding tissue and organs [[3], [4], [5]]. Studies show that the extent of the planned surgical procedure remains unchanged in half of rectal, gastric and duodenal GIST patients, despite neoadjuvant therapy completion [[6], [7], [8], [9], [10]]. This highlights the importance of early response monitoring and adequate selection of patients for neoadjuvant TKI treatment.

In clinical routine, response to TKI therapy is monitored by consecutive contrast-enhanced computed tomography (CE-CT) imaging. Several radiological criteria (RECIST 1.1, volumetry, and Choi), measuring changes in tumour characteristics over time (e.g., size and density), have been proposed to monitor treatment response in GIST patients [11]. According to ESMO guidelines (2022), a treatment duration of 6–12 months is recommended and resectability is re-evaluated when a stagnation in tumour shrinkage is observed [12]. However, the proposed duration of treatment is based on results from studies evaluating the use of TKI therapy in advanced metastatic GIST patients [[13], [14], [15], [16]]. The optimal duration of neoadjuvant treatment in non-metastatic GIST patients is not widely studied. Accurate and early response assessment is imperative to prevent surgical delay, overtreatment, side-effects, and costs in futile treatment.

This study aims to determine the effect of neoadjuvant TKI treatment on obtaining surgical benefit in non-metastatic GIST patients and evaluate whether these findings are correlated with radiological response assessment criteria (RECIST 1.1, volumetry and Choi). Furthermore, the temporal changes in tumour characteristics, as reflected by the radiological response criteria were evaluated. The value of these criteria to predict surgical benefit at an early stage was evaluated.

## Methods

### Patients

A total of 58 patients with confirmed non-metastatic primary GIST were referred for neoadjuvant TKI treatment or follow-up at the Leiden University Medical Center from October 2003 until April 2022 and retrospectively included. Inclusion criteria comprised patients receiving neoadjuvant TKI treatment, followed by curative-intended surgical resection and monitored using bi-phasic contrast enhance (CE)CT imaging. From the 58 patients, nineteen were excluded due to: missing imaging data (e.g., no biphasic imaging at baseline, no digital archiving data) (*n* = 11), concurrent treatment for a second malignancy (*n* = 2) or no resection (*n* = 6). Patients refrained from surgery, due to personal preference, comorbidities, or if the continuation of TKI treatment was preferred over a colostomy. Consequently, 39 GIST patients were included for final analysis. The study was conducted in accordance with the Declaration of Helsinki and approved by the local ethics committee (protocol code: B19.050, date of approval 14 January 2020). Requirement to obtain patient consent was waived due to the retrospective nature of the study.

### Surgical benefit

Included patients were evaluated for surgical benefit by two surgical oncologists with over twenty years of surgical experience in the field of GISTs (HHH, JvdH). They were asked to evaluate whether neoadjuvant TKI therapy led to de-escalation of surgical strategy (e.g., tissue and organ preservation) or reduced surgical complexity as regards to the baseline situation. Corresponding argumentation on the surgical benefits was also registered. The surgeons were provided access to all imaging and clinicopathological records (reporting on pathology, radiology, surgery, multidisciplinary meetings, etc.) and were blinded for the radiological assessments and reports in this study. Assessment was performed independently by the two surgeons, and disagreements were discussed in a consensus reading.

### Image acquisition and tumour segmentation

Biphasic CE-CT imaging was performed at baseline, during neoadjuvant treatment, and prior to surgery. The median interval between consecutive CE-CT examinations was three months. Baseline CE-CT examinations were obtained from different centres, whereas follow-up imaging was performed in a single academic centre. Details on the CT-acquisition parameters is provided in the Supplementary Materials. The portal-venous CE-CT scans with the smallest slice thickness (range 0.5 – 5.0 mm) were used for segmenting the primary tumour. Tumour segmentation was performed by a technical physician (YAW) using 3D slicer (version 5.0.2) [17]. Tumours were manually delineated on several axial slices and a slice-to-slice interpolation was used to automatically fill slices to create three-dimensional (3D) volumes of interest (VOIs). Segmentation was supervised by an abdominal radiologist with over 20 years of experience (AJvdM). The portal venous CE-CT scans of a random selection of twenty patients (ten baseline and ten preoperative scans) were independently delineated by a medical researcher (GMK) to assess interobserver variability.

### Radiological response assessment

Percentage changes in tumour density, volume and diameter between consecutive CE-CT scans were automatically determined for each 3D tumour volume using Python (version 3.7) [18]. Radiological response was determined between baseline and last preoperative portal venous CE-CT scans and evaluated according to RECIST 1.1, Choi, and volumetric criteria.

#### RECIST 1.1

The sum of the longest transaxial tumour diameters of five target lesions (SLD), evaluation of non-target lesions, and other oncological findings are used to determine treatment response. In this localised GIST population, the number of target lesions was limited to one primary tumour and no metastasis developed during TKI treatment. Therefore, changes in the longest transaxial tumour diameter was used to define the following types of treatment response; complete response (CR; disappearance of the primary tumour), partial response (PR; ≥30 % reduction in tumour diameter), progressive disease (PD; ≥20 % increase in tumour diameter) and stable disease (SD; neither progressive disease nor partial response) [19]. For the sake of consistency, the RECIST committee recommends measuring the longest diameter in the axial plane [20].

#### Volumetry

Volumetric response was defined as follows; CR (disappearance of the primary tumour), ≥66 % volume reduction (PR) and ≥73 % increase (PD) and SD (neither PD nor PR). The thresholds for volumetric response were based on thresholds for unidimensional change according to RECIST 1.1 and the relation to isotropic change in volume (as defined by a sphere).

#### Choi criteria

The Choi criteria, considers tumour diameter and tumour density. The following response categories can be defined; CR (disappearance of all lesions), PR (≥10 % reduction in tumour diameter or ≥15 % reduction in tumour density), PD (≥10 % increase in tumour diameter and <15 % reduction in tumour density) and SD (neither PD nor PR) [21].

### Statistical analysis

Given the small effect size, the Mann-Whitney *U* and Fisher's exact tests were performed for continuous data and categorical data, respectively. The median and interquartile range (IQR) were used to summarise data presented in this study. *P*-values below 0.05 were considered statistically significant. Cohen's kappa was used to measure the interobserver agreement between the two surgeons in the surgical benefit assessment. The reliability amongst different observers regarding tumour VOIs was assessed using the intra-class correlation coefficient (ICC) and percentage overlap as reflected by the Dice Similarity Coefficient (DSC). The correlation between radiological response criteria and surgical benefit was established by determining the accuracy, sensitivity, and specificity.

## Results

### Patient characteristics

Patient demographics and clinicopathological characteristics of the included patients are listed in Table 1. Patients initially received a daily imatinib mesylate dosage of 400 mg, except for one patient who received avapritinib (300 mg daily). Dosage was reduced in case of intolerable side-effects (*n* = 1) or reduced kidney function (n = 1) and escalated in patients with disease progression under treatment (n = 1) or low imatinib plasma levels (*n* = 2). The median interval between start of treatment and the last response scan was 8.3 months (IQR, 3.9–10.6). In a subset of 30 patients, interim response scans were performed over the course of treatment. The amount of interim response CE-CT scans varied from one to four per patient, with a total of 54 CE-CT scans. A median interval of 51 days (IQR, 5.5–73.0) was observed between the last response scan and surgery. TKI therapy was terminated just before surgery, which resulted in a median treatment interval of 9.9 months (IQR, 6.6–12.2).

**Table 1:** Patient characteristics. Numerical data is presented as median, along with the concurrent interquartile ranges (IQR). Tumour size was in this case defined by the largest tumour dimension. Risk stratification was assessed using the Miettinen classification system involving mitotic index, tumour site and location as prognostic factors. In four patients, the biopsy specimens were too small for a reliable mitotic count and risk stratification was therefore not reported. Neoadjuvant treatment was still administered in these patients, as tumours were relatively large (∼10 cm), or their location was difficult to access surgically (curvature minor of the stomach and gastroesophageal junction). An underlying PDGFRA 18 D842V mutation was observed in three patients and one patient was therefore treated with avapritinib (300 mg). (GI = gastrointestinal, PDGFRA = platelet-derived growth factor alpha, KIT = receptor tyrosine kinase).

	Total (*n* = 39)		Surgical benefit (*n* = 22)		No surgical benefit (*n* = 17)		p-Value
Age (years)	62.0	(54.0–71.0)	62.0	(46.0–66.5)	68.0	(55.0–72.0)	0.18
Sex							0.17
Female	12	(30.8 %)	9	(40.9 %)	3	(17.6 %)	
Male	17	(69.2 %)	13	(59.1 %)	14	(82.4 %)	
Tumour site							1.0
Stomach	29	(74.4 %)	18	(81.8 %)	11	(64.7 %)	Upper vs. lower GI tract
Duodenum	4	(10.3 %)	1	(4.5 %)	3	(17.6 %)	
Small intestine	3	(7.7 %)	1	(4.5 %)	2	(9.1 %)	
Rectum	3	(7.7 %)	2	(9.1 %)	1	(5.9 %)	
Largest primary tumour diameter (mm)	106.0	(79.5–206.0)	105.5	(85.0–209.0)	110.0	(72.0–139.0)	0.40
Risk stratification							1.00
Low	7	(17.9 %)	4	(18.2 %)	3	(17.6 %)	Moderate/high vs. low risk
Moderate/high	16	(41.0 %)	16	(4.5 %)	12	(70.6 %)	
Not reported	4	(10.3 %)	2	(9.1 %)	2	(9.1 %)	
Mutational status							0.16
KIT exon 11	28	(71.8 %)	18	(81.8 %)	10	(58.8 %)	KIT exon 11 vs. other mutations
KIT exon 9	2	(5.1 %)	1	(4.5 %)	1	(5.9 %)	
Wildtype	1	(2.6 %)	0	(0.0 %)	1	(5.9 %)	
PDGFRA exon 14	1	(2.6 %)	0	(0.0 %)	1	(5.9 %)	
PDGFRA exon 18	7	(17.9 %)	3	(13.6 %)	4	(23.5 %)	
D842V	3		1		2		
Non-D842V	4		2		2		
Interval start therapy-last scan (months)	8.3	(3.9–10.6)	9.1	(6.8–11.1)	5.2	(2.6–8.3)	0.01^a^
Treatment interval	9.9	(6.6–12.2)	10.7	(9.1–12.7)	7.7	(4.5–10.5)	<0.01^a^

a Upper gastrointestinal tract (stomach and duodenum), lower gastrointestinal tract (small intestine and rectum).

Comparison between the surgical-benefit and non-benefit group showed no statistical difference in age, sex, and tumour characteristics. However, the interval between the start of TKI treatment and the last preoperative scan, together with the total treatment interval were significantly higher in patients with surgical benefit. The same trend was observed within the subset of patients.

### Surgical benefit

**Fig. 1:**
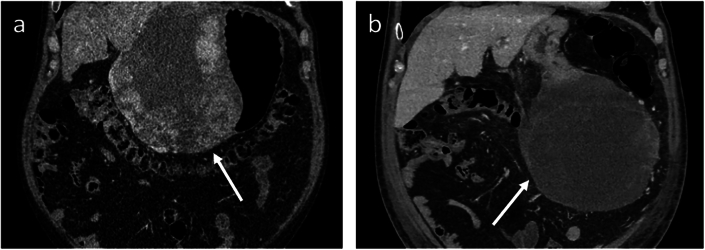
Coronal portal venous phase CE-CT images of a patient diagnosed with a gastric GIST (white arrows). Risk stratification of the primary tumour was not feasible, as no reliable mitotic count could be retrieved from the biopsy material. a) On the baseline CE-CT, the origin of the tumour was difficult to assess, as there is a great overlap between the GIST and the stomach. As a result, the surgeon would have initially opted for a partial gastrectomy. b) Preoperative CE-CT image after 1.7 months of TKI treatment (avapritinib), where the tumour dimensions remained relatively unchanged. The improved visualisation of the area of tumour attachment to the lesser curvature of the stomach is imperative for surgical decision making, as a partial gastrectomy could be avoided. Surgical strategy was de-escalated, and this patient was considered to have gained surgical benefit.

**Fig. 2:**
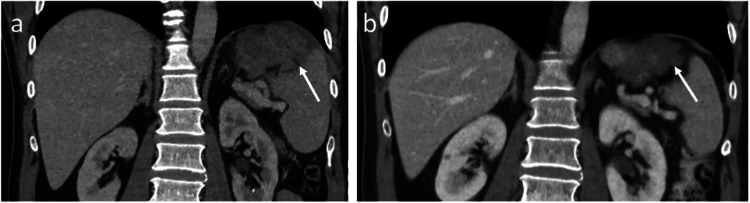
Coronal portal venous phase CE-CT images of a patient diagnosed with a high-risk gastric GIST (white arrows). a) On baseline CE-CT the tumour seemed adherent to the spleen and the left hemidiaphragm, which would suggest the need for a splenectomy. b) The preoperative CE-CT, showed a reduction in tumour size after 11.7 months of neoadjuvant TKI treatment (imatinib). The tumour no longer appeared adherent the spleen and left hemidiaphragm. Surgical benefit was gained in this patient, as TKI therapy facilitated spleen-sparing surgery.

**Fig. 3:**
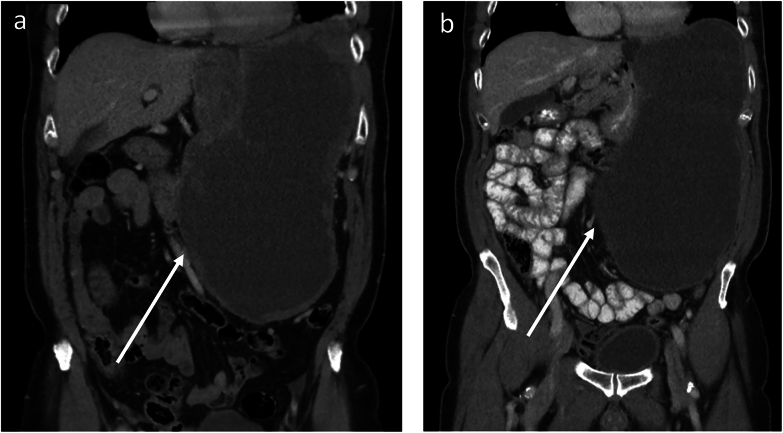
Coronal portal venous phase CE-CT images of a patient diagnosed with a large high-risk gastric GIST (white arrows). a) On baseline CE-CT imaging, the tumoural site of origin was difficult to assess and the tumour was adherent to the left hemidiaphragm. b) The preoperative CE-CT image. After 5.2 months of TKI treatment (imatinib), minimal tumour shrinkage was observed, and the visualisation of the tumour attachment was not improved. During the surgical procedure, the diaphragm was partially opened because of the tumour adherence to the left hemidiaphragm. During the process of tumour detachment, the tumour ruptured, causing potential iatrogenic tumour exposure to the dissection field. This patient did not benefit from TKI treatment, as there was no de-escalation in surgical strategy and surgical complexity was not reduced.

**Fig. 4:**
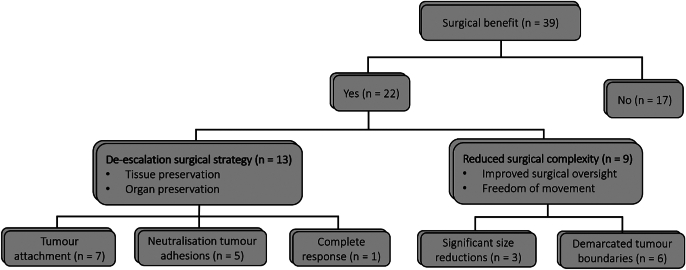
Flow diagram representing the surgical benefit assessment conducted by two expert oncological surgeons. They identified several factors contributing to the de-escalation of surgical strategy, including improved visualisation of tumour attachment, neutralisation of tumour adhesions and complete therapy response. Additionally, significant size reductions and more clearly demarcated tumour boundaries contributed to reduced surgical complexity.

Initial agreement between the two oncological surgeons on assessing surgical benefit was reached in 30 out of 39 of the included GIST patients (κ = 0.54). After the consensus reading, surgical benefit was reported in 56 % of patients (*n* = 22). The oncological surgeons identified several factors contributing to the de-escalation of surgical strategy, including improved visualisation of tumour attachment and neutralisation of tumour adhesions. In six patients, the surgeons opted for a local excision instead of a partial gastrectomy, due to smaller area of tumour attachment to the curvatures of the stomach (Fig. 1). Similarly, in one patient, a total gastrectomy was de-escalated to a partial gastrectomy. TKI treatment also facilitated neutralisation of tumour adhesions to adjacent organs. Consequently, organ-preserving surgery (e.g., spleen and anal sphincter) was feasible in five patients (Fig. 2). In one patient with a cardiac GIST, the primary tumour was undetectable during intraoperative inspection, and no resection was performed. In addition to less extensive surgical resections, the complexity of the surgical procedure was also reduced for several patients. Size reduction in initially large intra-abdominal tumours and well-demarcated tumour boundaries both provided greater surgical oversight and freedom of movement, thereby reducing surgical complexity. Surgical procedures for large intra-abdominal tumours or tumours growing from complex anatomical sites (e.g., lesser curvature of the stomach, pelvic region, horizontal part of the duodenum) adjacent to critical structures are more challenging. In three patients, large intra-abdominal tumours reduced significantly in size over the course of treatment. In six other patients, tumours were more clearly demarcated from their surroundings. Ultimately, TKI therapy did not induce any of the abovementioned surgical benefits in seventeen patients (Fig. 3). A flow diagram of the surgical benefit assessment is provided in Fig. 4.

### Radiological response assessment

There was high interobserver reliability between the segmentations made by the two observers, as reflected by a high DSC of 0.92 (±0.06 standard deviation). Furthermore, there was high interobserver reliability regarding tumour size, volume and density with an ICC value over 0.99 [22]. Response status was determined for all patients according to RECIST 1.1 (18 PR, 20 SD and 1 PD), volumetric criteria (20 PR, 19 SD) and Choi criteria (34 PR, 4 SD and 1 PD).

### Surgical benefit reflected by radiological response criteria

Results of the surgical benefit assessment were compared to the radiological response criteria determined between baseline and last preoperative CE-CT scans. The accuracy, sensitivity, and specificity for Choi and RECIST1.1 were (64 %, 24 %, 96 %) and (90 %, 100 % 82 %) respectively. Volumetry had the highest performance scores (95 %, 100 % and 91 %).

**Fig. 5:**
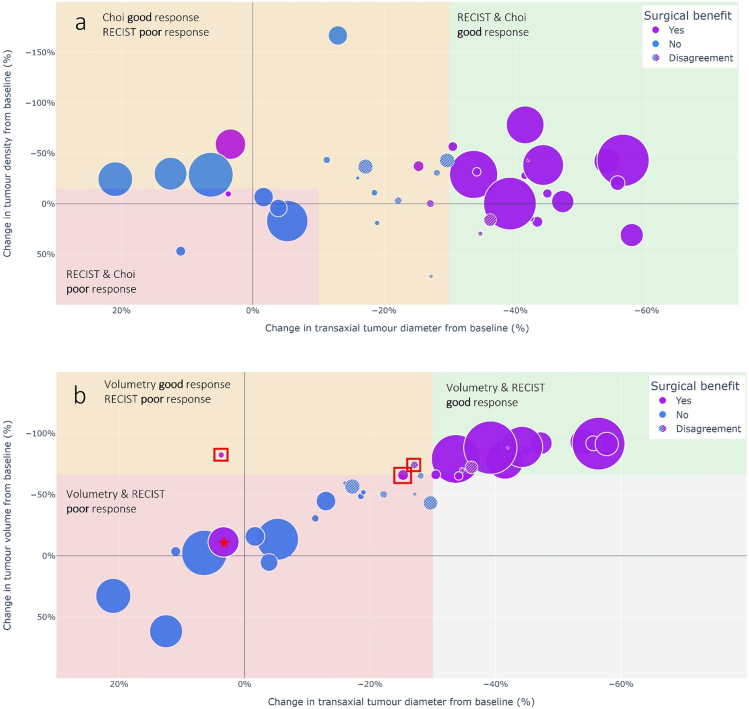
a) Bubble plot visualising the percentage change in transaxial tumour diameter against the percentage change in tumour density. b) Bubble plot visualising the percentage change in transaxial tumour diameter against the percentage change in tumour density. Negative values denote a decrease in size or density. Each bubble represents an individual non-metastatic GIST patient treated with neoadjuvant TKI treatment. The bubble colour resembles the assessment on surgical benefit (magenta = surgical benefit, blue = no surgical benefit, striped filling = initial disagreement), while the bubble size represents the scaled initial tumour volume at diagnosis. The grids have been divided into three parts by using the thresholds presented in current radiological response criteria (red = agreement on bad response, green = agreement on good response and orange = discrepancies on responder status). Nine patients that the surgeons initially disagreed on during the surgical benefit assessment (striped filling), were all close to predefined size thresholds (−30.0 % in diameter and −66.0 % in tumour volume). Three patients were classified as non-responders by the RECIST criteria, while the percentage change in tumour volume was above 66.0 % and surgical benefit was observed (red boxes). In one misclassified patient, TKI therapy was considered surgically beneficial, while the volume only reduced with 10.8 % (★). Improved visualisation of the area of tumour attachment was the decisive factor for determining surgical benefit (see Fig. 1 for patient case). (For interpretation of the references to colour in this figure legend, the reader is referred to the web version of this article.)

The effect of TKI treatment on surgical benefit was adequately reflected by the radiological criteria that based their response status on tumour size measurements (RECIST 1.1 and volumetry) compared to density measurements (Choi). Fig. 5 summarises the correlation between radiological tumour characteristics and findings on surgical benefit. Noteworthy is that in patients where initial assessment on surgical benefit resulted in disagreement, reductions in tumour diameter and volume were close to the predefined thresholds, with a median absolute deviation of 4.7 % (IQR, 2.8–7.8 %) and 9.4 % (IQR, 6.3–16 %).

**Fig. 6:**
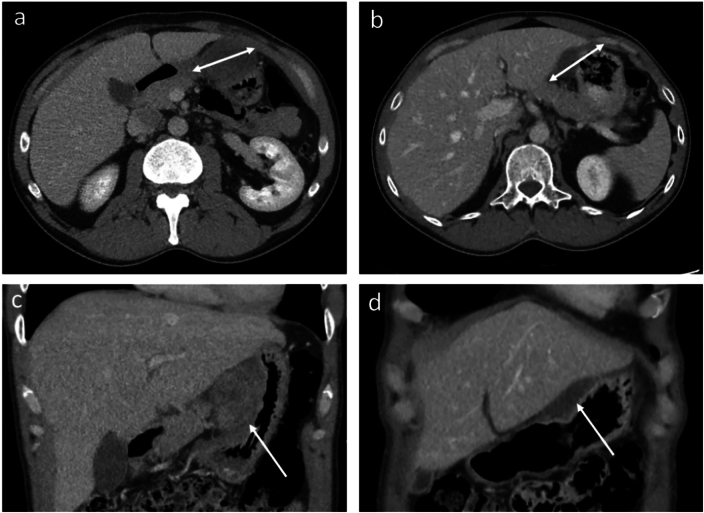
a-b) Axial portal venous phase CE-CT images of a patient diagnosed with a low-risk gastric GIST. a) Baseline CE-CT image where the transaxial tumour diameter measured 59 mm. b) Preoperative CE-CT image after 6.7 months of TKI treatment. The transaxial tumour diameter equalled 62 mm, which would suggest tumour growth rather than tumour shrinkage. c-d) However, when looking at the coronal portal venous CE-CT images, a significant decrease in tumour volume (arrows) can be observed between the c) baseline CE-CT image and d) final preoperative CE-CT image. The percentage change in tumour volume equalled 82 %.

In this study, RECIST 1.1 classified three patients as stable disease, whereas the reduction in tumour volume was above 66.0 %. Besides, surgical benefit was still achieved in these patients (Figs. 5b and 6). One patient was misclassified by both RECIST 1.1 and volumetric criteria. A small reduction in tumour volume (11 %) improved overall visualisation of tumour attachment significantly, which was considered crucial for surgical decision-making (Figs. 5b and 1).

### Temporal change in tumour size over the course of treatment

**Fig. 7:**
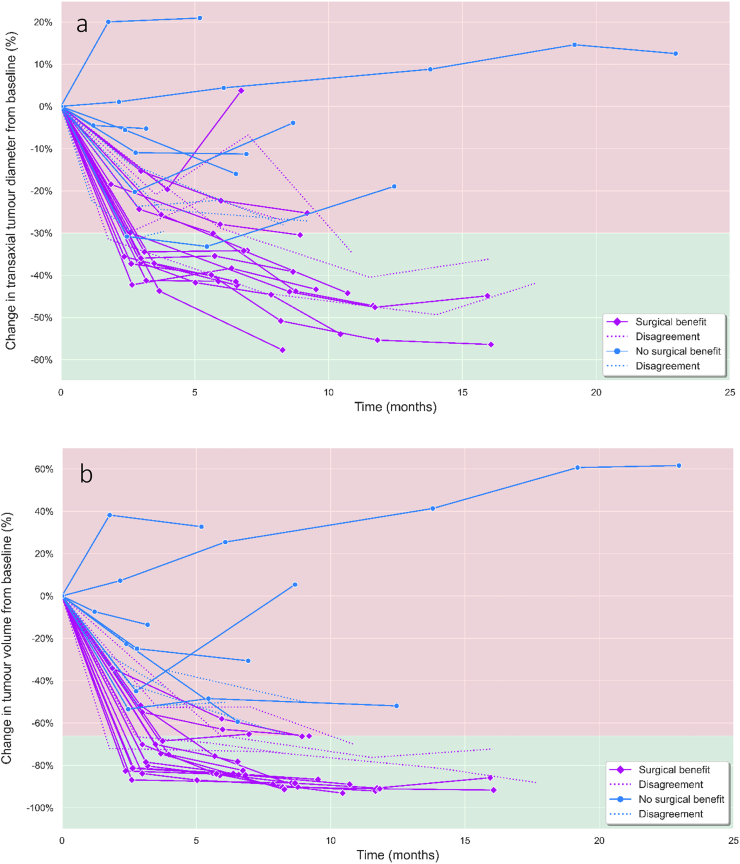
a) Spaghetti plots visualising the individual percentage change in a) transaxial tumour diameter and b) tumour volume over the course of TKI treatment. This analysis was executed on a subset of patients (*n* = 30), who were monitored using additional interim response portal venous CE-CT scans, next to the fixed baseline and preoperative scans. Each line represents a non-metastatic GIST patient, and the colours resemble the concurrent assessment on surgical benefit (magenta = surgical benefit, blue = no surgical benefit). Dashed lines represent patients that surgeons initially disagreed on during surgical benefit assessment. The grids have been divided into two parts by using the predefined thresholds (−30.0 % in diameter and −66.0 % in tumour volume) as presented in current radiological response criteria (green = good response, red = poor response). Tumour progression was observed in three patients with moderate/high risk GISTs of the ileum and stomach, all harbouring KIT exon 11 mutations. One of these patients was treated for 23.1 months, since they initially refrained from surgical resection. (For interpretation of the references to colour in this figure legend, the reader is referred to the web version of this article.)

Fig. 7 shows the trend in tumour size over the course of TKI treatment. Out of 30 patients, nineteen were considered to have gained surgical benefit, while eleven did not benefit from neoadjuvant treatment. Accuracy, sensitivity, and specificity for predicting surgical benefit after the first CE-CT interim response for RECIST 1.1 (68 %, 91 % and 53 %) and volumetric criteria (83 %, 100 %, and 74 %) were computed.

Median time between baseline imaging and the first interim scan was 2.5 months (IQR, 2.0–2.8) in the non-benefit group and the median reduction in volume was 25 % (IQR, 15–43 %). This was significantly less (*p* < 0.01) when compared to a median reduction of 72 % (IQR, 66–80 %) in the surgical benefit group at an interval of 3.0 months (IQR, 2.6–3.5). All nineteen surgical-benefit patients eventually reached a volume reduction above 66 %, whereas in fourteen patients this threshold was already reached after the first interim response scan. During the second response scan (median follow-up 6.6 months) tumours reduced only slightly in volume with a median of 3.1 % (IQR, 2.1–7.8 %). This stagnation in tumour shrinkage was less apparent when assessing response using unidimensional RECIST measurements, as depicted by Fig. 7a.

## Discussion

In this study, the incidence of surgical benefit after neoadjuvant TKI treatment in GIST, as determined by two specialised surgical oncologists, was studied. Furthermore, the efficacy of radiological response criteria (i.e., RECIST 1.1, Choi, and volumetry) to predict response to neoadjuvant TKI treatment for obtaining surgical benefit was investigated.

In 56 % of patients, response to TKI was classified as surgically beneficial by two oncological surgeons. These findings are in line with reported incidence in previous literature [8]. Most frequent causes contributing to surgical benefit were improved visualisation of tumour attachment and demarcated tumour borders. Initial consensus between surgeons was 77 %, reflecting the subjective and complicated nature of assessment of surgical benefit. Although surgical benefit was accurately reflected by size-based radiological criteria (RECIST 1.1 and volumetry), we observed one patient that was considered to have obtained surgical benefit, while they were classified as non-responder by both RECIST 1.1 and volumetric criteria. This demonstrates that surgical benefit is not only determined by quantitative reductions in tumour size, but also by more subjective findings involving the presence of tumour adhesions and visualisation of tumour attachment.

Although the Choi criteria have been successfully used to monitor changes in tumour vascularity in GIST by measuring the percentage change in attenuation coefficients, size-based criteria (RECIST 1.1 and volumetry) were more strongly correlated with surgical benefit. One of the factors that could have contributed to variability in Choi criteria, are non-uniform acquisition and reconstruction protocols used for CT imaging. These variations could have had a significant impact on the attenuation coefficients [[23], [24], [25]]. Since baseline imaging is often performed before tertiary referral, heterogeneity in acquisition protocols exists in clinical routine, making it difficult to prevent concurrent variability in tumour density measurements. Besides, changes in vascularity may show signs of response, but it does not necessarily reflect surgical benefit. The heterogeneity in acquisition protocols and limited clinical predictive power to determine surgical benefit, make the Choi criteria less practical and precise for assessment of neoadjuvant treatment-induced surgical benefit.

Volumetry more accurately predicted whether patients would have gained surgical benefit after neoadjuvant treatment when compared to unidimensional diameter measurements. It was observed that the effect of neoadjuvant TKI treatment can be underestimated using only the largest unidimensional diameter, particularly if the reduction in tumour size is not isotropic (i.e., significant reduction across a single dimension). Schiavon et al. also showed that volume criteria classified a higher number of patients as partial responders compared to RECIST 1.1, which is in accordance with our findings [26]. Although this study shows that volumetry might be preferred when monitoring response to neoadjuvant treatment in GIST, practical limitations often prevent the use of such a measure in routine clinical practice. In order to limit the amount of additional labour required for tumour segmentation and improve repeatability, the use of automatic segmentation algorithms can facilitate the use of volumetric response metrics in clinical practice [27].

To the author's knowledge this is the first study investigating the relation between radiological response criteria and surgical benefit obtained after neoadjuvant treatment. Significant reductions in tumour size were observed within a time interval of 3 months. This is in line with another study, where biggest reduction in tumour load (as determined by RECIST 1.1) in metastatic GIST patients, was observed at 3.5 months [16]. However, Tirumani et al. observed the best volumetric response in non-metastatic GIST patients after a longer follow-up, namely 6.5 months [15]. The difference in follow-up might be explained by a difference in methodological approach. Tirumani et al. determined the best response through a volumetric threshold of 40 %, as proposed in Graser et al. [28,29]. Furthermore, tumour volumes were approximated by the ellipsoid equation, rather than segmenting the tumour, making estimations less accurate.

In patients where surgical benefit was obtained, initial substantial reductions in tumour size were followed by a stagnation in tumour shrinkage. This phenomenon has also been reported in literature [15,16,30]. Because tumour size reductions are associated most with surgical benefit, it is questionable whether patients gain any additional surgical benefits beyond this point. This observation poses an important question on whether neoadjuvant treatment should be stopped at an earlier time point. Considering treatment related side-effects, such an early decision could have significant impact on patient quality of life. However, it should be noted that a significant number of surgical-benefit patients also received adjuvant TKI treatment, potentially limiting the benefit of such an early decision.

Given the limited population size and retrospective nature of this study, results should be interpreted with caution. Patients that refrained from surgery were excluded from analysis, as surgical benefit could not be assessed without surgical reports. This may have introduced selection bias, especially in some patients where TKI treatment was preferred over surgery, suggesting relatively good response. The median treatment interval is significantly greater in the surgical benefitting group, which may have been a confounding factor. Nevertheless, minimal to no size reductions were observed after three months of treatment.

## Conclusion

This study shows that using size-based radiological response criteria based on CE-CT imaging could accurately predict surgical benefit achieved by neoadjuvant treatment in non-metastatic GIST patients. Using tumour volumetry, patients where surgical benefit was obtained could already be identified within 3 months after treatment initiation.

The following is the supplementary data related to this article.

**Fig S1:**
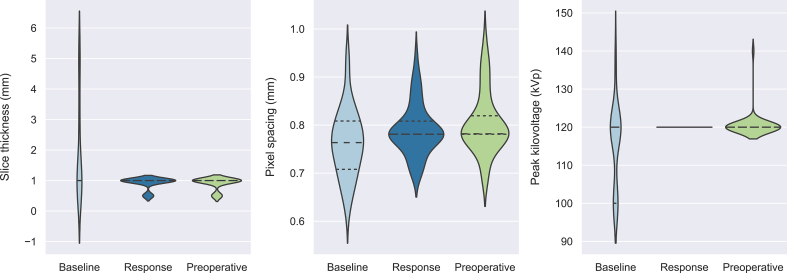
Violin plots visualising the distribution of acquisition parameters (slice thickness, pixel spacing and peak kilovoltage) for baseline (39 scans, 39 patients), interim response (54 scans, 30 patients), and preoperative (39 scans, 39 patients) portal venous CE-CT imaging. Baseline imaging was often performed in other hospitals, while response and preoperative imaging were both executed in a tertiary sarcoma referral centre. This has caused a heterogeneity within the acquisition protocols. The median value for slice thickness, pixel spacing and peak kilovoltage at baseline (1.0 mm, 0.76 mm, 120 kVp), response (1.0 mm, 0.78 mm, 120 kVp) and preoperative imaging (1.0 mm, 0.78 mm, 120 kVp) were computed. In baseline imaging (39 scans), four scanner manufacturers were observed including Siemens Healthineers, GE Healthcare, Canon Medical Systems (previously known as Toshiba Medical Systems) and Philips Healthcare. Response (54 scans) and final preoperative imaging (39 scans) were performed using only Canon Medical imaging systems.

## Institutional review board statement

The study was conducted in accordance with the Declaration of Helsinki, and approved by the Ethics Committee Leiden Den Haag Delft (METC LDD) (protocol code: B19.050, date of approval: 14 January 2020).

## Informed consent statement

Patient consent was waived due to the retrospective nature of the study. Patients who objected to the use of their data were excluded.

## CRediT authorship contribution statement

**Ylva A. Weeda:** Writing – review & editing, Writing – original draft, Visualization, Validation, Software, Resources, Project administration, Methodology, Investigation, Formal analysis, Data curation, Conceptualization. **Gijsbert M. Kalisvaart:** Writing – review & editing, Writing – original draft, Supervision, Software, Resources, Methodology, Investigation, Formal analysis, Data curation, Conceptualization. **Henk H. Hartgrink:** Writing – review & editing, Data curation, Conceptualization. **Aart J. van der Molen:** Writing – review & editing, Visualization, Data curation. **Hans Gelderblom:** Writing – review & editing. **Judith V.M.G. Bovée:** Writing – review & editing. **Lioe-Fee de Geus-Oei:** Writing – review & editing, Supervision, Resources, Methodology, Funding acquisition, Conceptualization. **Willem Grootjans:** Writing – review & editing, Writing – original draft, Visualization, Validation, Supervision, Resources, Methodology, Investigation, Formal analysis, Data curation, Conceptualization. **Jos A. van der Hage:** Writing – review & editing, Visualization, Supervision, Resources, Methodology, Investigation, Data curation, Conceptualization.

## Declaration of competing interest

The authors declare the following financial interests/personal relationships which may be considered as potential competing interests: We declare the following financial interests/personal relationships which may be considered as potential competing interests: G.M. Kalisvaart was the recipient of an educational grant (LEI-05) from Philips Electronics Nederland B.V, Eindhoven and supported by a public grant (LSHM18089) from Health Holland TKI Life Sciences & Health. The funders had no role in the design of the study, in the collection, analyses, or interpretation of data, in the writing of the manuscript or in the decision to publish the results.

## Data Availability

The datasets generated during and/or analysed during the current study are available from the corresponding author upon reasonable request.
